# West Nile Virus Vaccine Design by T Cell Epitope Selection: *In Silico* Analysis of Conservation, Functional Cross-Reactivity with the Human Genome, and Population Coverage

**DOI:** 10.1155/2020/7235742

**Published:** 2020-03-19

**Authors:** Frances M. Waller, Pedro A. Reche, Darren R. Flower

**Affiliations:** ^1^School of Life and Health Sciences, Aston University, Aston Triangle, Birmingham, UK B4 7ET; ^2^Immunomedicine Group, Facultad de Medicina, Departamento de Inmunologia & O2, Universidad Complutense de Madrid, Madrid, Spain

## Abstract

West Nile Virus (WNV) causes a debilitating and life-threatening neurological disease in humans. Since its emergence in Africa 50 years ago, new strains of WNV and an expanding geographical distribution have increased public health concerns. There are no licensed therapeutics against WNV, limiting effective infection control. Vaccines represent the most efficacious and efficient medical intervention known. Epitope-based vaccines against WNV remain significantly underexploited. Here, we use a selection protocol to identify a set of conserved prevalidated immunogenic T cell epitopes comprising a putative WNV vaccine. Experimentally validated immunogenic WNV epitopes and WNV sequences were retrieved from the IEDB and West Nile Virus Variation Database. Clustering and multiple sequence alignment identified a smaller subset of representative sequences. Protein variability analysis identified evolutionarily conserved sequences, which were used to select a diverse set of immunogenic candidate T cell epitopes. Cross-reactivity and human leukocyte antigen-binding affinities were assessed to eliminate unsuitable epitope candidates. Population protection coverage (PPC) quantified individual epitopes and epitope combinations against the world population. 3 CD8+ T cell epitopes (ITYTDVLRY, TLARGFPFV, and SYHDRRWCF) and 1 CD4+ epitope (VTVNPFVSVATANAKVLI) were selected as a putative WNV vaccine, with an estimated PPC of 97.14%.

## 1. Introduction

West Nile Virus (WNV) is a mosquito-borne Flavivirus that causes West Nile Fever (WNF) and West Nile neuroinvasive disease (WNND) in birds, humans, and horses [1]. Originating in the West Nile regions of Uganda in 1937, WNV has now become a prevalent human infection. Before mid-1990, the virus was confined to Africa and Europe, then spread to North America, the Middle East, and West Asia. Two and a half million cases were reported between 1999 and 2010, of which, 12,000 were WNND, resulting in over 1300 deaths. Thus, WNV has become a major global public health concern.

WNV infection manifests as one of three disease states: asymptomatic carrier, West Nile Fever (WNF), and West Nile neuroinvasive disease (WNND) [1]. After the initial mosquito bite, 3-14 days elapse before the first symptoms, with rapid progression thereafter. Asymptomatic carriers represent 75% of all cases, with 25% presenting with WNF or WNND [2]. WNF is a mild, self-limiting disease which presents as general fever, malaise, and muscle and gastrointestinal pain [3]. Overall mortality is ~4.2% but rises to 9.6% in WNND.

WNV is part of the Flavivirus genus, which comprises over 70 viruses and many human pathogens, including numerous mosquito-borne viruses [4]. WNV is transmitted from an infected host via a mosquito bite, primarily by the Culex spp. and to a lesser extent the Aedes spp. [5]. Human and equine infections occur outside the natural transmission lifecycle sustained between mosquitos and birds [6]. Horses and humans are “dead-end” hosts, unable to reinfect mosquitos due to insufficient viremia [4].

The WNV genome comprises a single-stranded nonsegmented positive sense RNA of ~11,000 nucleotides [7]. It is transcribed into a single polyprotein, which is cleaved by viral proteases into ten mature viral proteins: three 5′ structural segments (C, PrM, and E) and seven 3′ nonstructural protein elements (NS1, NS2A, NS2B, NS3, NS4A, NS4B, and NS5). Phylogenetic analysis suggests that there are at least seven distinct WNV lineages [8]. While infection from other lineages is known, major human outbreaks arise solely from lineages 1, 2, and 5 [6].

There are no current FDA-approved WNV treatments. Reducing exposure to mosquitos is the main strategy, via mosquito nets, protective clothing, and insect repellent and by staying indoors [8]. In the absence of viable therapeutic interventions, effective vaccines could provide long-term protection against WNV. No commercial human WNV vaccines exist [9], but successful vaccines for closely related Flaviviruses—Japanese encephalitis virus, tick-borne virus, and Yellow Fever virus—suggest that an effective, well-tolerated WNV vaccine is feasible [10]. Adaptive immune responses promote viral clearance and control WNV infection [11]. Several novel vaccine candidates are currently in phase I and phase II clinical trials [12], yet WNV clinical trials face several challenges including late or sporadic presentation of symptoms, asymptomatic carriers, inconsistency of outbreaks, trial logistics, and comorbidities in the elderly [8].

The only trialed WNV inactivated vaccine comprised a minimally pathogenic Kunjin virus incubated with hydrogen peroxide [9]. PrM and E proteins of lineages 1 and 2 have been exploited as subunit vaccines. Lineages 1 and 2 can provide crossprotection against other lineages suggesting that a single universal vaccine is feasible [13]. Recent approaches have tried to induce neutralising antibodies by targeting highly immunogenic E antigens. Capitalising on the successful equine vaccine PreveNile [12], the most promising current human vaccine, ChimeriVax-WN02, uses the Yellow Fever 17D backbone to incorporate PrM and E genes [10]. Three gene mutations (L107F, A316V, and K440R) attenuated the virus [13].

PrM, E, NS3, and NS4B proteins are commonly targeted by CTLs [11]. Long-lasting immunity has been achieved in phase I subunit vaccine trials using adjuvants and DIII regions of the WNV E protein [4]. A DNA vaccine expressing the NY99 capsid protein generates a strong CD4+ immune response with a significant rise in IL-2 and IFN-*γ* levels [12]. Vaccines expressing domain II of the E protein have produced WNV-neutralising antibodies in phase I trials [9].

The lack of extant WNV vaccines prompts us to evaluate potential epitope ensemble vaccines as an alternative, exploiting our evolving approach to vaccine design. We have exemplified this by identifying putative vaccines against hepatitis C [14], influenza [15], malaria [16], Epstein-Barr virus [17], TB [18, 19], and dengue [20]. By focusing on highly conserved immunogenic epitopes with a broad population coverage, we identified optimal selections of prevalidated epitopes of proven immunogenicity. To avoid undesired immunogenicity in designed vaccines, we extend our prior work here to filter out epitope cross-reactivity with the human genome.

## 2. Methods

### 2.1. Identification of West Nile Virus CD8+ and CD4+ T Cell Epitopes

Experimentally confirmed West Nile epitopes (ID: 11082) were retrieved from the Immune Database and Analysis Resource (IEDB) (http://www.iedb.org/). Epitopes were restricted to host “Humans,” “T cell Assays,” “Positive Assays,” and “Any MHC restrictions.” CD8+ and CD4+ data were collected separately.

### 2.2. Acquisition, Processing, and Alignment of the West Nile Virus Polyprotein

Complete West Nile Virus genome sequences for all human variants and lineages were obtained from the National Centre for Biotechnology Information (NCBI) West Nile Virus Variation Database Resource (URL: https://www.ncbi.nlm.nih.gov/genome/viruses/variation/WestNile/). Incomplete and duplicated sequences were removed. Sequences were clustered using CD-HIT (URL: http://weizhongli-lab.org/cdhit_suite/cgi-bin/index.cgi?cmd=cd-hit) to form a set of representative sequences. Identified cut-off was 0.99. The clustered sequences were multiply aligned using MUSCLE (URL: http://www.ebi.ac.uk/Tools/msa/muscle/) to form multiple sequence alignment (MSA).

### 2.3. Analysis of Epitope Sequence Variability

Conserved epitopes were identified by analysing conservation of the MSA, using the Protein Variability Server (PVS) [21] (URL: http://imed.med.ucm.es/PVS/). The first sequence in the alignment was used as a reference. Sequence variability was masked, and only fragments with a length greater or equal to 9 were selected. The Shannon entropy threshold was 0.5. CD8+ and CD4+ epitopes with at least 50% overlap were retained for subsequent analysis.

### 2.4. Analysis of Epitope Cross-Reactivity

CD8+ cross-reactivity was analysed using iCrossR (URL: http://webclu.bio.wzw.tum.de/icrossr/) after determining class I binding profiles, as below. The number of mismatches was set to “0, 1, 2, and 3” for each binding prediction. Epitopes with a cross-reactivity index (ICR) of 0.01 or greater were removed.

### 2.5. HLA Binding Profile Prediction and Calculation of Population Protection Coverage (PPC)

Binding affinities of conserved CD8+ (http://tools.iedb.org/mhci/) and CD4+ (http://tools.iedb.org/mhcii/) T cell epitopes were predicted using IEDB. HLA I reference set was used for MHC I epitopes (Weiskopf, Angelo et al. 2013) and an HLA II reference set was used for MHC II epitopes (Greenbaum et al. [22] Class I binding profiles present in the top one percentile rank were retained. For MHC Class II, epitopes less than 15 amino acids in length were eliminated and binding profiles in the top five percentile rank were obtained. Conserved CD8+ epitopes were also analysed using EPISOPT (URL: http://bio.med.ucm.es/episopt.html) selecting all ethnic groups in the US population (Caucasian, Black, Hispanic, Asian, and native North American) and a PPC above 95%. Global PPC values for highly-conserved epitopes were calculated using IEDB (http://tools.iedb.org/tools/population/iedb_input). MHC I and MHC II epitopes were then ranked by PPC. Epitopes were combined within each class to calculate overall PPC values. To create a potential ‘universal' vaccine candidate CD8+ and CD4+ epitopes were combined and PPC calculated using IEDB as above.

## 3. Results

Searching IEDB identified 165 linear CD4+ and CD8+ T cell epitopes presented to T cells during WNV infection: 53 HLA class I and 112 HLA class II epitopes. Epitope length ranged from 8 to 20 amino acids. Genomic sequences representing all strain variants of WNV were also retrieved from the NCBI West Nile Virus Variation Database. 126 unique protein sequences were retrieved. AJR27178, AJR27181, and AJR27181; AJW82677 and AKH144860; and AJW59216 and AJW59220 were found to be identical. Only unique sequences were retained. Sequence clustering using CD-HIT identified nonredundant sequences representative of all WNV sequences. All major human lineages were present; 5 sequences were generated with two representing lineage 2: AJW59217 (USA, 2002), AJR27898 (Italy, 2014), AHB37632 (Italy, 2013/08), AMZ00438 (India, 1988/02/12), and ALK02494 (Australia, 1991). AHB37632 was discarded due to sequence anomalies.

A multiple sequence alignment (MSA) was performed on the remaining 4 sequences. All four lineages were highly conserved: 3069 positions had identical amino acids (89.37%), and only 64 positions showed variable amino acids (1.87%). The remainder was either partially conserved (3.17%) or highly conserved (5.59%). Analysis with PVS showed that 122 of the 165 epitopes had ≥50% sequence identity to the masked WNV reference sequence: 32 CD8+ 9mers, three 10mers, and one 11mer, with 19 epitopes showing 100% sequence identity; 86 CD4+ epitopes had ≥50% sequence identity, with 19 of the 86 epitopes showing 100% sequence identity.

As therapeutic peptides can evoke autoimmune responses, conserved epitopes were screened computationally to detect undesirable cross-reactivity against human tissues. Cross-reactivity (CR) was computed using iCrossR for HLA-I 9mer epitopes only. The output for each mutation was averaged to calculate an overall cross-reactivity index (ICR).

Our results show that none of the epitopes have an ICR greater than 0.01; thus, it seems unlikely that self-antigen recognition and toxic effects would occur; when tested at the three mutation levels, many epitopes did exhibit CR. HLA-I binding profiles and PPC were calculated using EPISOPT and IEDB. All CTL 9mer epitopes were entered into EPISOPT to estimate the PCC for the five US ethnic groups (see Table 1). ITYTDVLRY had the largest number of binding alleles (10) and the highest PPC. EPISOPT analysis showed that a PPC > 95% could be reached using HLA-I alleles alone.

Using three epitopes, the maximum PPC was 97.65%, with 24 distinct class I restrictions: TLARGFPFV, GPIRFVLAL, and ITYTDVLRY. 190 different combinations of four epitopes achieved a PPC > 95%. TLARGFPFV and ITYTDVLRY were present in most highly scoring epitope sets, with many epitopes absent from all candidate ensembles.

HLA-I binding profiles were also obtained using IEDB. Peptides in the top 1% rank were retained to ensure strong binding and sufficient immunogenicity. More HLA-A binding alleles (14) were seen than HLAB (9). The IEDB PPC tool predicted that the top EPISOPT ensemble had only an 84.68% PPC. To achieve a PPC of >95%, 6 epitopes are needed: TLARGFPFV, ITYTDVLRY, KSYETEYPK, SYHDRRWCF, MPNGLIAQF, and GPIRFVLAL (see Table 2).

HLA-II binding profiles were estimated using IEDB, retaining epitopes in the top 5% rank. PPC calculation showed that individual epitopes had a relatively high PPC, with 8 having values over 50%. VTVNPFVSVATANAKVLI and GEFLLDLRPATAWSLYAV had the highest value: 70.55%. ILVSLAAVVVNPSVKTVR and VTVNPFVSVATANAKVLI achieved a combined PPC of 81.81%. The addition of further epitopes had no effect. Many CD4+ epitopes had binding profiles that were subsets of other epitopes. These epitopes were removed, leaving a set of HLA-II alleles covering all epitopes (see Table 3). Many of the high-scoring epitopes also had overlapping binding profiles.

Both CD4+ and CD8+ T cells are important in viral clearance. CD8+ and CD4+ epitopes were combined to calculate a PPC, using the PPC tool on IEDB. By combining the top two epitopes from each HLA subset VTVNPFVSVATANAKVLI and ILVSLAAVVVNPSVKTVR, and ITYTDVLRY and TLARGFPFV, a PPC of 96.36% was achieved. A vaccine ensemble comprising ITYTDVLRY, TLARGFPFV, and SYHDRRWCF and VTVNPFVSVATANAKVLI covered 97.14% of the world's populations. This increased to 99.52% when using 11 epitopes (see Table 4).

## 4. Discussion

West Nile Virus has been recognized as a reemerging global pathogen. Present in Africa for over 50 years, recent geographical transmission has raised its profile as a public health concern. There are no current effective treatments, and the cost-to-benefit ratio of the WNV development pipeline is poor. Vaccination is a key intervention. An efficacious WNV vaccine could significantly benefit the global population. Vaccines are available for closely related Flaviviruses and against equine WNV, resulting in a significant reduction in annual mortalities. Veterinary vaccine Equilis West Nile is an inactivated whole virus vaccine comprising a strain known as Yellow Fever-West Nile. The vaccine is given to horses over six months via 2 intramuscular injections, 3 to 5 weeks apart, with a single booster injection given a year later. In comparative evaluations, correlates of protection were seen in 89-94% of treated animals in different test groups. Most previous candidate WNV vaccines have relied on B cell-mediated immunity. Here, we attempt to identify highly conserved T cell epitopes that might form an epitope ensemble sufficiently immunogenic to protect against geographically diverse WNV strains.

Epitopes in adoptive immunotherapies may exhibit undesired side effects [23], such as CR when foreign peptide sequences resemble those of self-peptides sufficiently to initiate an unwanted autoimmune response. Addition of computational CR prediction to our design-by-selection protocol is a key advance over previous work [14–20] and should accelerate the early selection of safe vaccines. When using iCrossR [23], none of the epitopes were identified to elicit responses cross-reactive with human tissues. McMurtrey et al. [24] and Kaabinejadian et al. [25] identified epitopes presented by HLA-A∗02:01 and HLA-A∗11:01, and many were also selected by our approach, including RVL9, SVG9, TLA9, KYS9, AVV9, RLD10, ATW9, and SLT9. YTM9, SLF9, and KNM9 were eliminated as they lacked ≥50% sequence identity to the viral reference.

No single epitope provided protection against WNV. A PPC > 95% was only possible when multiple epitopes were combined. A potent immune response needs both CD4+ and CD8+ T cell responses [26]. Combined epitopes generate greater T cell responses than a single epitope, reinforcing the need for multiple conserved epitopes [27]. At least four epitopes were needed for a PPC > 95%. HLA class I 9mers (TLA9, SYH9, and ITY9) combined with one HLA-II epitope (VTV18n) gave a cumulative population coverage of 97.14%.

Virus replication introduces mutations which can eventually generate new strains or alter existing ones; yet, we found that 90% of the WNV sequence is conserved (*H* < 0.5). This lack of variability is an aid to vaccine design as conserved regions can be exploited as therapeutic targets, as newly emerging strains retain conserved amino acids. CD-HIT was used to remove redundant protein sequences. While remaining sequences represented the most common human WNV lineages (1a, 1b, 2, and 5), the less common lineages (3 and 4) were excluded. To generate a truly universal vaccine, crossprotection against all WNV lineages and strains should be investigated. Thus, a second-generation WNV vaccine may need inclusion of lineages 3 and 4.

All T cell epitopes included in our final vaccine combination were either located in the E protein (VTV18), NS2A (ITY9), NS3 (SYH9), or NS4B (TLA9). Finding highly conserved epitopes in the E protein, NS3, and NS4B is expected, since previous work has shown these proteins to be highly immunogenic and common targets for CTLs [11, 28]. Variability across the genome is uneven, with the structural proteins being the least variable [29].

The C region has the highest proportion of residue alterations (~23%) [30]. The E protein has been exploited in previous vaccine design: most current DNA vaccine candidates against Flaviviruses express the viral E and PrM proteins [31]. Sarri and coworkers identified HLA alleles responsible for susceptibility to WNV infection. They categorized HLA alleles to be either “protective,” increased “susceptibility,” or CNS-high risk [32]. None of the protective binding alleles suggested by Sarri et al. were identified here. This “protective” function of HLA alleles could be exploited in WNV vaccine development to provide protection for all ethnicities.

By considering functional cross-reactivity with human proteins, the work described here represents a step forward in our evolving approach to vaccine design. Work based on naive sequence similarity have not previously proved useful [33–35]. Here, it proved possible to combine 4 epitopes—3 CD8+ and 1 CD4+ T cell epitopes—to achieve a global PPC of 97.14%. Combined CTL and CD4+ are required for successful viral clearance. The ensemble identified is a viable starting point for further in vitro characterization or phase 0 trials, since we can assume that this epitope selection is likely both safe and immunogenic in the majority of most populations. This paper emphasises the application of computational cross-reactivity prediction to vaccine design, only allowing selection of epitopes without either structural or sequence similarity to the human genome. Overall, our work provides a promising starting point for the exploration of next-generation WNV vaccines.

## Figures and Tables

**Table:** (a) EPISOPT

Peptide	HLA-I binding profile EPISOPT	PPC (%)
ITYTDVLRY	A0301 A1101 A6801 B1502 B1516 B1517 B2702 B2709 B5502 C0702	44.66
TLARGFPFV	A0201 A0202 A0203 A0204 A0205 A0206 A6802	34.52
SLFGGMSWI	A0201 A0202 A0206 B1508	27.58
KSYETEYPK	A1101 A3101 A3301 A6801	14.86
LTYRHKVVK	A0301 A1101 A3101 A3301 A6801 B1502	32.51
RVLSLIGLK	A0203 A0301 A1101 A3101	24.47
AVVVNPSVK	A0301 A1101	17.83
SYHDRRWCF	A2402 C0702	22.05
RYLVKTESW	B1513 B5701 B5702	2.48
MPNGLIAQF	B0702 B1502 B1508 B3501 B3801 B5301 B5401 B5702 B5801	35.45
GPIRFVLAL	A0203 A0214 B0702 B0801 B3501 B5301 B5401 C0304	44.48
AEVEEHRTV	A2902 B4002	7.11
WMDSTKATRY	—	0
KGDTTTGVY	B1516 B5702 B5801	2.13
VVEKQSGLY	A1101	4.70
QTDNQLAVF	A0207 B3801 B5702 B5801	3.71
ALRGLPIRY	A0201 A0203 A0301 B1517	27.34
TEVMTAVGL	A2902 B39011 B3909 B4002 B4402	12.41
MTTEDMLEVW	—	0
RPAADGKTV	B0702 B5101 B5102 B5103 B5401 B5502	18.59
ILRNPGYAL	A0202 A0206 A0214 B0702 B1502 C0304	29.62
RVLEMVEDW	B1513 B5701 B5702 B5801	5.79
RSLFGGMSW	B5701	1.93
RAWNSGYEW	B5801	1.59
HTTKGAALM	B1510	0
SVGGVFTSV	A0201 A0202 A0203 A0206	28.88
VLNETTNWL	A0201	18.04
SLVNGVVRL	A0201 A0202 A0203 A0205 A0206 A0214 B1510 B1517 B3909	34.47
FVDVGVSAL	A0204 A0205 A0206 A0207 A0214 B3801 C0102	6.65
YRHKVVKVM	B1510 B2701 B3801 B39011 B3909	3.94
RRSRRSLTV	B2703 B2704 B2705 B2706 B2709	2.39
ATWAENIQV	A0202 A0204 A0205 A0207 A0209 B1516	1.49

**Table:** (b) IEDB

Peptide	HLA-I binding profile IEDB (top 1 percentile rank)	PPC (%)
ITYTDVLRY	A0101 A0301 A3002 B5801 B5701 A2601 A1101	54.74
TLARGFPFV	A0206 A0203 A0201	41.35
SLFGGMSWI	A0203 A0201	39.84
KSYETEYPK	A1101 A0301 A3001 A3101	38.48
LTYRHKVVK	A0301 A1101 A3101	35.36
RVLSLIGLK	A0301 A1101 A3001	34.14
AVVVNPSVK	A1101 A0301	30.92
SYHDRRWCF	A2301 A2402	26.18
RYLVKTESW	A2301 A2402	26.18
MPNGLIAQF	B5301 B3501 B0702	22.88
GPIRFVLAL	B0702 B0801	22.61
AEVEEHRTV	B4403 B4001 B4402	20.88
WMDSTKATRY	A0101 A3002	19.55
KGDTTTGVY	A3002 A0101	19.55
VVEKQSGLY	A3002 A0101	19.55
QTDNQLAVF	A0101	17.34
ALRGLPIRY	A0301	16.81
TEVMTAVGL	B4001 B4403	13.83
MTTEDMLEVW	B5701 A6802 A0206 B5301 B5801	13.7
RPAADGKTV	B0702	12.78
ILRNPGYAL	B0702	12.78
RVLEMVEDW	B5701 B5801 A3201	11.53
RSLFGGMSW	B5701 B5801 A3201	11.53
RAWNSGYEW	B5701 B5801	7.26
HTTKGAALM	A2601	5.82
SVGGVFTSV	A6802 A0203	3.46
VLNETTNWL	A0203	0.97

**Table 2:** Candidate epitope ensemble vaccines with >95% PPC, as calculated by EPISOPT and IEDB.

Ensemble	Method	Peptides		PPC
1	EPISOPT	TLARGFPFV ITYTDVLRY	GPIRFVLAL	99.00

2	EPISOPT	TLARGFPFV ITYTDVLRY	GPIRFVLAL MPNGLIAQF	99.00

3	EPISOPT	TLARGFPFV TEVMTAVGL MPNGLIAQF	ITYTDVLRY	99.23

4	EPISOPT	TLARGFPFV ITYTDVLRY	GPIRFVLAL	97.65

5	IEDB	TLARGFPFV KSYETEYPK MPNGLIAQF AEVEEHRTV MTTEDMLEVW RSLFGGMSW	ITYTDVLRY SYHDRRWCF GPIRFVLAL	97.34

6	IEDB	TLARGFPFV KSYETEYPK MPNGLIAQF AEVEEHRTV MTTEDMLEVW	ITYTDVLRY SYHDRRWCF GPIRFVLAL	96.81

7	IEDB	TLARGFPFV KSYETEYPK MPNGLIAQF AEVEEHRTV	ITYTDVLRY SYHDRRWCF GPIRFVLAL	96.49

8	IEDB	TLARGFPFV KSYETEYPK MPNGLIAQF GPIRFVLAL	ITYTDVLRY SYHDRRWCF	95.25

**Table 3:** Subset of epitopes that represent all HLA-II alleles by elimination of repetitive binding profiles.

Epitope	HLA-II binding profile top 5 percentile rank	PPC
VTVNPFVSVATANAKVLI	HLA-DRB1∗08:02, HLA-DRB1∗04:01, HLA-DRB1∗09:01, HLA-DRB1∗11:01, HLA-DRB1∗04:05, HLA-DRB1∗01:01, HLA-DRB3∗02:02, HLA-DRB5∗01:01, HLA-DRB1∗07:01, HLA-DRB1∗13:02, HLA-DRB1∗15:01, HLA-DQA1∗01:02/DQB1∗06:02	70.55
ILVSLAAVVVNPSVKTVR	HLA-DRB1∗08:02, HLA-DRB1∗01:01, HLA-DRB1∗03:01, HLA-DRB1∗04:01, HLA-DQA1∗01:02/DQB1∗06:02, HLA-DRB1∗12:01, HLA-DRB1∗04:05, HLA-DRB1∗15:01, HLA-DRB1∗13:02, HLA-DQA1∗05:01/DQB1∗03:01, HLA-DRB1∗11:01, HLA-DRB3∗02:02	69.22
RVVFVVLLLLVAPAYS	HLA-DPA1∗03:01/DPB1∗04:02, HLA-DPA1∗02:01/DPB1∗01:01, HLA-DRB1∗11:01, HLA-DPA1∗01:03/DPB1∗02:01, HLA-DPA1∗01/DPB1∗04:01, HLA-DRB1∗12:01, HLA-DRB1∗08:02, HLA-DRB1∗15:01, HLA-DRB1∗04:01, HLA-DRB1∗04:05, HLA-DPA1∗02:01/DPB1∗05:01, HLA-DRB1∗01:01, HLA-DRB3∗01:01, HLA-DRB5∗01:01, HLA-DRB1∗03:01, HLA-DQA1∗05:01/DQB1∗02:01	65.33
SGNVVHSVNMTSQVLLGR	HLA-DRB1∗03:01, HLA-DRB1∗07:01, HLA-DRB1∗04:05, HLA-DRB1∗04:01, HLA-DRB1∗11:01, HLA-DQA1∗01:02/DQB1∗06:02, HLA-DRB1∗08:02, HLA-DRB1∗13:02, HLA-DRB4∗01:01, HLA-DRB1∗04:01	59.32
VLSLIGLKRAMLSLIDGK	HLA-DRB1∗08:02, HLA-DRB1∗11:01, HLA-DRB1∗09:01, HLA-DRB5∗01:01, HLA-DRB1∗03:01, HLA-DPA1∗02:01/DPB1∗14:01, HLA-DRB4∗01:01, HLA-DRB3∗02:02, HLA-DRB1∗15:01, HLA-DPA1∗02:01/DPB1∗01:01	49.41
LVQSYGWNIVTMKSGVDV	HLA-DRB1∗07:01, HLA-DRB5∗01:01, HLA-DQA1∗01:01/DQB1∗05:01, HLA-DRB1∗15:01	34.78
GLLGSYQAGAGVMVEGVF	HLA-DRB1∗09:01, HLA-DQA1∗04:01/DQB1∗04:02, HLA-DQA1∗03:01/DQB1∗03:02, HLA-DRB1∗07:01, HLA-DQA1∗01:02/DQB1∗06:02, HLA-DQA1∗05:01/DQB1∗03:01, HLA-DRB1∗01:01	34.02

**Table 4:** Final epitope combinations corresponding to candidate epitope ensemble vaccine and PPC.

Ensemble	HLA-I epitopes	HLA-II epitopes	PPC (%)
1	ITYTDVLRY TLARGFPFV	VTVNPFVSVATANAKVLI ILVSLAAVVVNPSVKTVR	96.36
2	ITYTDVLRY TLARGFPFV SYHDRRWCF	VTVNPFVSVATANAKVLI	97.14
3	ITYTDVLRY TLARGFPFV KSYETEYPK	VTVNPFVSVATANAKVLI	95.23
4	ITYTDVLRY TLARGFPFV KSYETEYPK SYHDRRWCF	VTVNPFVSVATANAKVLI ILVSLAAVVVNPSVKTVR	98.71
5	ITYTDVLRY TLARGFPFV KSYETEYPK SYHDRRWCF MPNGLIAQF	VTVNPFVSVATANAKVLI ILVSLAAVVVNPSVKTVR	99.01
6	ITYTDVLRY TLARGFPFV KSYETEYPK SYHDRRWCF MPNGLIAQF GPIRFVLAL AEVEEHRTV MTTEDMLEVW RSLFGGMSW	VTVNPFVSVATANAKVLI ILVSLAAVVVNPSVKTVR	99.52

## Data Availability

Data is available from http://www.iedb.com and available from the authors.
